# The Relationship Between In-Play Betting and Gambling Problems in an Australian Context of Prohibited Online In-Play Betting

**DOI:** 10.3389/fpsyt.2020.574884

**Published:** 2020-10-23

**Authors:** Sally Melissa Gainsbury, Brett Abarbanel, Alex Blaszczynski

**Affiliations:** ^1^Brain and Mind Centre, School of Psychology, The University of Sydney, Darlington, NSW, Australia; ^2^International Gaming Institute, University of Nevada, Las Vegas, Las Vegas, NV, United States

**Keywords:** in-play betting, live action betting, regulation, online gambling, internet gambling, problem gambling, disordered gambling, gambling addiction

## Abstract

Internationally, Internet gambling is increasingly permitted under regulated licensing conditions; however, the specific products that are legal varies between jurisdictions. Online sports and race wagering are now legal in many jurisdictions, but in-play betting (also referred to as “live action” or “in-the-run” betting) is often restricted. In-play betting enables bets to be placed on an event after it has commenced. Prohibitionist policies often cite the potential for this type of betting to increase risk of gambling problems. This study aimed to identify which online bettors are most likely to engage in in-play betting, and to investigate the relationship between in-play betting and gambling problems. Online survey responses were collected from 501 Australian past-month online sports bettors in the context of in-play betting only being available on offshore gambling sites or via telephone betting. Thirty-four percent of participants had placed a bet in-play in the past month. Participants placing in-play bets differed from those who had not in terms of education, employment status, ethnicity, age, and gambling involvement. Those who bet in-play had higher problem gambling severity scores than those who did not bet in-play. Problem gambling severity significantly predicting in-play betting, holding other variables constant. Findings are consistent with previous research indicating that the relationship between in-play gambling and problems holds across jurisdictions which have prohibited and legalized in-play betting. The findings suggest that in-play betting should warrant specific regulatory attention and interventions to minimize gambling harms among individuals that engage with this activity.

## Introduction

In-play betting (also termed “live,” “live action,” or “in-the-run” betting) refers to betting markets that allow bets to be wagered after an event, such as a race or sporting matches, have commenced. In-play betting is becoming an increasingly popular feature of contemporary gambling markets (1). Statistics on the prevalence of in-play betting are scarce as it is common for the activity not to be specifically measured in prevalence studies. However, one UK-based survey conducted in 2018 found that 45.4% of bettors surveyed (aged 18–54 years) had bet in-play (2). Another UK study found that in-play betting among 18- to 24-year-olds rose from 38% in 2015 to 45% in 2016 (3). In-play betting, combined with online and mobile betting availability, has fueled the growth of sports betting. For example, tennis is the third most profitable market for betting companies despite its relatively low fan base. Eighty percent of wagers on tennis events are reportedly placed after the match begins (4).

It is important for policy makers to understand the impact of specific gambling activities on harms to guide regulatory approaches to minimizing gambling problems. The structural features of in-play betting, including the short delay between the bet and the outcome, the small window for decisions to place a bet, variability in outcomes, and continuous options for bets are speculated to contribute to gambling-related harms (5). This paper aims to increase understanding of the types of individuals participating in in-play betting and to explore the potential association between this betting activity and gambling problems. Greater understanding of in-play betting and the subgroup of individuals who engage in this activity is essential to inform policy decisions and design targeted interventions to enhance well-being and minimize potential harms associated with in-play betting.

### The Regulatory Context

Gambling policy has a significant impact on the rates of harm experienced within communities. For example, jurisdictions with less stringent regulations regarding advertising of online gambling have higher rates of sub-clinical disordered gambling (6). There are three main types of in-play betting: (i) betting on the final outcome of an event after it has started (e.g., which team will win a sporting match); (ii) exotic wagering, betting on contingencies that may or may not happen during the course of an event (e.g., a specific player will score the next goal in a football game); and (iii) micro-betting, betting on a subset of an event (e.g., the outcome of the next point in a tennis match) (7, 8).

Internationally, the United Kingdom and Italy allow in-play betting (including micro-betting), whereas France prohibits micro-betting but allows in-play betting on match outcomes and some forms of exotic wagering (e.g., goals scored, goal scorers) (8). In the United States, gambling and betting is legislated at the state level, so in-play betting (commonly referred to as proposition or “prop” bets) rules differ across the nation and mode of access permissions follow general sports betting rules. In Nevada, for example, in-play betting is permitted for a variety of wagers, such as point spreads, money lines, and totals (9). Online betting is permitted following in-person registration, which involves initial account deposits made in-person at a sportsbook or registered location (10). In Iowa, in-play wagering is permitted except for wagers involving in-state collegiate teams (11). Online betting is permitted from anywhere in the state once an individual has registered in-person at a casino [a requirement that ends in 2021; (11)].

In Australia, online in-play sports betting is prohibited under the Interactive Gambling Act (12) (IGA); however, in-play race and sports betting are permitted when bets are placed on-site (in a venue) or over the telephone [i.e., by way of voice calls to customer service centers, but not VoIP (Voice over Internet Protocol) or “click to call” where consumers use handheld Internet-connected devices to speak to an automated system to place their bets]. This approach was intended to reduce the risk to individuals vulnerable to experiencing gambling problems, particularly for higher risk variants of in-play betting (e.g., those involving very short-term, repetitive betting). Various arguments have been made for the legalization of online in-play betting in addition to on-site and telephone in-play betting (essentially, platform neutrality). These arguments typically relate to the ability of licensed operators to compete with offshore gambling providers and to the increasingly obsolete distinction between online and telephone in-play betting (7, 13).

Interviews conducted with community respondents and industry and sporting body stakeholders indicate that most in-play betting within Australia occurs in sports due to the opportunities to place bets on a greater range of outcomes as compared to racing (1). Online in-play betting has raised issues in relation to sports integrity because the outcomes of subsets of events, as opposed to the event itself, can be manipulated relatively easily (7, 8).

### Structural Characteristics of Online In-Play Betting

Structural characteristics of gambling—inherent features of games—can contribute to the acquisition, maintenance, and development of problem gambling behaviors (14). Structural features of online in-play betting may have greater potential for causing gambling-related harm than telephone or in-venue in-play betting. Online in-play betting has been likened to “continuous gambling” (5, 15). Continuous forms of gambling are characterized by a short duration of time between the bet being placed and the outcome becoming known, providing a structure that allows gamblers to immediately reinvest money in a rapid sequence, resulting in fast and repetitive betting (16, 17). The rapid speed of play tends to encourage more bets, longer gambling sessions, loss-chasing, and impaired self-control (18). Furthermore, the nature of in-play betting means that there is limited time to make the decision about placing a bet. An experimental French study found that participants used more heuristic than analytical processes when placing bets under time constraints, theoretically leading to less reasonable bets (19). However, there remains very limited empirical and ecologically valid research to justify claims regarding the potential of online in-play betting to cause gambling-related harm.

### The Association Between Online In-Play Betting and Problem Gambling

A series of studies have been conducted using customer account data on *bwin*, a predominately European gambling site. Live action online sports betting was the only form of gambling associated with potential gambling-related problems when assessments were made on screen-based activity and after controlling for participation in another 15 gambling activities (20). These results are confirmed by several separate analyses of customer data. In the first month after opening an account, customers characterized by high intensity and frequency of live action gambling and by high variability of wager sizes were more likely to report gambling-related problems upon account closure than other customers who placed live action bets (21). Greater intensity of gambling activity, such as a greater number of bets placed per day, appears to clearly distinguish customers who trigger a responsible gambling alert from controls, particularly in relation to live action sports betting (22). Customers who played at least two games and demonstrated high variability in live action wager amounts were identified as a high-risk group when controlling for first deposit date (23). Participating at least three times in live action betting was a significant predictor of increased risk of experiencing gambling-related problems after controlling for involvement in multiple gambling activities (24). Although these studies provide some evidence of a link between in-play betting and gambling problems, several relied on proxy indicators of gambling harm or subsets of customers reporting problems, thereby not capturing all gamblers experiencing problems. Additionally, these studies provide limited insight into the typical characteristics of individuals placing in-play bets, such as severity of gambling problems, associated gambling activity, and demographic factors.

A prospective longitudinal study of Internet sports gamblers from 85 countries found that participants betting in-play on sports, relative to those betting before matches, were categorized more often as heavily involved gamblers (25). The prototypical bettor was a 31-year old male betting for longer periods of time than females. Data from the UK gambling prevalence survey indicates that online gamblers who bet in-play are more likely to be classified as having a gambling problem and are at greater risk of harm from gambling than those who do not bet in-play (26). In a Spanish sample of sports gamblers, in-play betting was more prevalent among those with a gambling problem than any other group (27). Furthermore, those with a gambling problem bet more heavily in-play compared to before games commenced. Analysis of customer account data from a small sample of individuals classified as having gambling problems found that live betting increased betting opportunities and motivated loss chasing, resulting in persistent and extended betting sessions (28). In-play betting created fewer natural breaks in play due to short periods between a bet being placed and the outcome being determined, thus reducing the opportunity for reductions in arousal and other emotional responses stimulated by betting, winning, and losing.

An Australian study of 1,816 sports bettors found that men aged between 18 and 34 years were most likely to have participated in in-play betting (29). More highly engaged bettors, including those with gambling problems, were more likely to bet on micro events, and were more likely to place a higher proportion of their bets on micro events (15). Micro-event bettors tended to be younger, well-educated, single, and to have high trait impulsivity. They engaged in a higher number of different gambling forms in addition to sports betting, bet on a higher number of different sports, had more accounts with different operators, and used a higher number of different sports betting promotions. Of those who bet on micro events, 78% met the criteria for problem gambling, whereas only 5% met the criteria for non-problem gambling (vs. 29 and 28%, respectively, for non-micro event bettors). Moreover, placing a higher proportion of bets on micro-events was related to problem gambling. Within an Australian sample, respondents were more likely to bet on in-play sporting events than on pre-match outcomes if they were characterized by having higher trait impulsivity, more frequent sports betting behavior, higher problem gambling severity and a shorter history of sports betting (30). However, these studies either focus on a subset of in-play betting (15) or examine sports betting across an aggregation of online, telephone, and retail betting channels (15, 29–31) and several of the separately published results are based on the same dataset, limiting the differential conclusions drawn.

Taken together, these studies indicate that intensity and frequency of live action sports betting is associated with gambling-related problems among individuals who place online bets. However, many of the previous studies fail to control for overall gambling involvement and use proxy behaviors as indicators of level of harm, making it difficult to ascertain the extent to which in-play betting is predictive of current or future experience of gambling harm. Given that online in-play betting may be associated with gambling-related harms over and above that of telephone or on-site in-play betting (15, 18), it is imperative to examine the relationship between problem gambling and online in-play betting specifically.

### The Current Study: Aims and Hypotheses

This study aimed to understand the association between online in-play betting and gambling problems in the context of online in-play betting being prohibited on licensed domestic gambling sites. Specifically, the study sought to determine: (i) the proportion of regular online gamblers who engage in in-play sports betting; (ii) the characteristics this sub-group; and (iii) whether there is an association between online in-play betting and increased risk for gambling problems. The findings contribute to existing knowledge concerning participation in online in-play betting and clarify whether individuals who participate in online in-play betting are at increased risk of experiencing gambling problems. Moreover, this research is needed to inform international policy debates regarding the legalization of online in-play betting. Given the relative lack of research on this area, the study was largely exploratory. However, we hypothesized that use of in-play wagering would be associated with higher problem gambling severity.

## Methods

Recruitment occurred using market research online panel sampling. To participate, respondents had to be 18 years of age or older and have gambled online during the past 4 weeks. Potential respondents received an email from the market research company providing a brief outline of the study and a URL to access the online questionnaire. Participation was voluntary and respondents could withdraw at any time. Ethics approval for this research was received from the [deidentified] University Human Research Ethics Committee.

A total of 1,001 were responses collected and our initial sample was a subset of *N* = 501, consisting of all participants who indicated they had wagered on sports during the prior 4 weeks. Respondents were mostly male (67.8%), married (52.6%), and employed full-time (53.6%). Age ranged from 18 to 83, with a significant difference in mean age for males (*M* = 45.5, *SD* = 14.8) and females (*M* = 38.1, *SD* = 12.7), *t*_(362.98)_ = 5.74, *p* < 0.001, *d* = 0.53.

### Measures

#### Gambling Frequency and Behaviors

Fixed choice questions assessed frequency of spending real money on seven types of Internet gambling activities: lottery-type games, slot machines, race wagering, esports betting^1^, sports betting, poker, casino card or table games, and other. Response options were at least once per day, at least once per week, or at least once in the last 4 weeks. Respondents were also asked to indicate (yes/no) if they had placed a wager on an event after it had started (i.e., an in-play bet). Questions assessed age at which participants had first gambled and modes used to place bets (smartphone, computer, tablet, wearable device, telephone, in venue).

#### Demographics

Age, gender, education, work status, family household income, language spoken at home, country of birth, and ethnic background.

#### Gambling Problems

The nine-item Problem Gambling Severity Index (PGSI) (32) assessed the extent of gambling-related harm experienced over the previous 12 months. Total scores range from 0 to 27 and are used to classify respondents into the following categories: non-problem gambling (PGSI = 0), low-risk gambling (PGSI = 1–2), moderate-risk gambling (PGSI = 3–7), and problem gambling (PGSI = 8–27). Cronbach's alpha in this sample was 0.95. The PGSI has been independently validated and shown to have excellent reliability, dimensionality, external/criterion validation, item variability, practicality, applicability, and comparability (33).

### Statistical Analysis

The data were analyzed using SPSS 26.0. Assumptions testing was conducted on all measured variables, including skewness and kurtosis, univariate outliers, and multivariate outliers (Mahalnobis distance). Where instances of homogeneity of variance is violated, a Satterthwaite approximation for degrees of freedom is applied. One multivariate outlier was found and removed from the database, resulting in a sample of *N* = 500 for further analysis. Age first gambled was highly skewed and leptokurtic, which was corrected with a log transformation. Missing values for the in-play betting variable were excluded on a list wise basis.

Chi-square tests and *t*-tests were used to investigate if group differences existed between sports bettors who participate in in-play betting and those who do not for single-response demographic and gambling behavior variables. Following these comparisons, a logistic regression was conducted to determine which characteristics differentiate in-play bettors from non-in-play bettors. Twelve predictor variables were used in the logistic regression: gender, age, education level, employment status, income, ethnic background, country of birth, language other than English spoken at home, number of gambling behaviors (other than sports betting), age first gambled, highest reported gambling frequency for any gambling game, and PGSI classification (binary variable, classified as problem gambling for scores of 8 or higher). These variables were selected based on established validity from other studies [see, e.g., (34–36)].

For comparison testing, an alpha of 0.05 was used and effect sizes are reported for all *t*-tests and chi-squares. For *t*-tests, Cohen's *d* is reported (small effect = 0.2, medium effect = 0.5, and large effect = 0.8). For chi-square comparisons, the ϕ (phi) coefficient was used (small effect = |0.1|, medium effect = |0.3|, and large effect = |0.5|). Where measurement of certain variables is not conducive to certain analytical procedures (i.e., questions offered multiple response options and thus percentage responses sum to more than 100%), these frequency percentages are provided without statistical comparisons. Following the omnibus tests, standardized residuals (±2) were examined to determine where cell differences lie.

## Results

Just over one third of the participants (34.4%) reported having placed a wager on an event after it had started (i.e., participated in in-play betting) during the prior 4 weeks and were classified as in-play bettors.

### Demographics

As shown in Table 1, participants who bet in-play were significantly younger than those who did not bet in-play; those aged 50 years and over particularly more likely to not bet in-play than those under the age of 40 years, χ^2^ (4, *N* = 500) = 42.80, *p* < 0.001, ϕ = 0.29. Participants that bet in-play were statistically more also likely to have completed higher education levels (e.g., university or college degree, post graduate qualification) χ^2^ (3, *N* = 500) = 24.45, *p* < 0.001, ϕ = 0.22. In terms of employment status, a higher proportion of participants that bet in-play were employed full-time, and a lower proportion were the recipient of welfare, χ^2^ (3, *N* = 500) = 28.89, *p* < 0.001, ϕ = 0.24. There were no significant differences in terms of reported household income. Participants who bet in-play were more likely to be of Asian or Middle Eastern backgrounds than those who did not, who were more likely to be from European backgrounds, χ^2^ (3, *N* = 500) = 40.70, *p* < 0.001, ϕ =0.29. In-play betting participants were more likely to speak a language other than English at home, χ^2^ (1, *N* = 500) = 10.55, *p* < 0.001, ϕ =0.15, although there was no significant difference in country of birth (*p* >0.05). The difference in gender proportions between groups approached significant levels, but was not statistically significant (*p* = 0.053).

**Table 1:** Comparison of the demographic profiles of participants who bet in-play vs. those who did not bet in-play (*N* = 500).

**Demographic characteristic**	**In-play betting** **(*N* = 172) (%)**	**No in-play betting** **(*N* = 328) (%)**
**Gender**		
Male	62.2	70.7
Female	37.8	29.3
*p* = 0.053 (*χ^2^* = 3.75, df = 1)		
**Age**		
18–19	2.3	1.2
20–29	27.3	12.5
30–39	33.1	22.6
40–49	22.7	24.1
50 and over	14.5	39.6
*p <* 0.001 (*χ^2^* = 42.80, df = 4)		
**Education**		
Year 12 or equivalent	24.4	31.7
Trade/technical certificate/diploma	16.9	31.7
University or college degree	43.6	25.6
Post graduate qualification	15.1	11.0
*p* < 0.001 (*χ^2^* = 24.45, df = 3)		
**Employment status**		
Work full time	65.3	48.6
Work part time or casual	20.0	17.3
Non-salaried	10.0	10.8
Welfare recipient	4.7	23.2
*p* < 0.001 (*χ^2^* = 28.89, df = 3)		
**Family household annual income**		
< $25,000	6.3	5.6
$25,000–$49,999	16.5	23.8
$50,000–$74,999	17.7	17.2
$75,000–$99,999	19.6	18.8
$100,000–$124,999	15.2	13.9
$125,000–$149,999	14.6	9.6
$150,000–$174,999	4.4	3.6
$175,000–$199,999	2.5	3.6
$200,000 or more	3.2	4.0
*p* > 0.05		
**Country of birth**		
Australia	80.2	84.5
Not Australia	19.8	15.5
*p* > 0.05		
**Language other than English**		
Yes	18.0	8.2
No	82.0	91.8
*p* = 0.001 (*χ^2^* =10.55, df = 1)		
**Ethnic origin**		
European	57.0	79.6
Asian (including East, Southeast, and South Asian)	30.2	10.1
Middle Eastern	4.7	1.2
Other	8.1	9.1
*p* < 0.001 (*χ^2^* = 40.70, df = 3)		

### Gambling Involvement

Table 2 displays reported gambling behaviors and history. In terms of game preference, the most popular form of gambling among participants who bet on sports was lottery-type games. Participants that bet in-play engaged in all forms of gambling at a higher frequency than those who did not bet in-play, with a notably large difference for esports betting (58.7 vs. 17.7%), poker (59.3 vs. 28.0%), and casino card or table games (61.6 vs. 28.7%). Participants that bet in-play engaged in 4.17 (*SD* = 1.97) of the six additional reported forms of gambling (not including sports betting, for which the entire sample indicated they had played) in the past 4 weeks, which was significantly higher than the 2.55 (*SD* = 1.86) mean forms for those who did not place in-play bets, *t*_(493)_ = 9.02, *p* < 0.001, *d* = 0.84.

**Table 2:** Comparison of gambling behaviors and history of profiles of participants who bet in-play vs. those who did not bet in-play (*N* = 500).

	**In-play betting** **(*N* = 172) (%)**	**No in-play betting** **(*N* = 328) (%)**
**Games played (past 4 weeks involvement)**		
Lottery-type games	81.4	68.3
Slot machines, pokies, electronic gaming machines	76.7	50.3
Esports betting	58.7	17.7
Race wagering	79.1	64.0
Poker	59.3	28.0
Casino card or table games (not including poker)	61.6	28.7
Highest gambling frequency*		
At least once per day	36.0	13.4
At least once per week	54.1	68.3
At least once in the last 4 weeks	9.9	18.3
*p* < 0.001 (*χ^2^* = 36.04, df = 2)		
**Years of age when first gambled**		
17 and under	6.4	14.5
18–19	32.6	41.0
20–29	44.2	29.9
30–39	13.4	8.6
40–49	2.3	3.1
50 and over	1.2	2.8
*p* = 0.002 (*χ^2^* = 18.98, df = 5)		

Chi-square values are not displayed where the question allowed multiple responses to be selected.

* *Highest gambling frequency taken as highest response to any form of gambling*.

Participants that bet in-play were more likely to gamble at least once per day [χ^2^ (2, *N* = 500) = 36.04, *p* < 0.001, ϕ = 0.27] and were more likely to be older (*M* = 23.12, *SD* = 6.78) when they first gambled compared to those who did not bet in-play (*M* = 22.02, *SD* = 7.87), χ^2^(5, N = 500) = 18.98, *p* = 0.002, ϕ = 0.20.

Participants that bet in-play had a significantly higher average PGSI score (*M* = 8.76, *SD* = 6.65) than those who did not bet in-play (*M* = 3.68, *SD* = 5.17), *t*_(281.76)_ = 8.72, *p* < 0.001, *d* = 0.85.

Of those who indicated that they had placed in-play bets, the most popular mode of access was using online websites and apps via smartphone (50.0%), followed by personal computer (48.8%), tablet (11.6%), and wearable device (2.9%), none of which are permitted by gambling sites licensed in Australia. In-play bettors placed their bets via legal, regulated modes of access, including speaking over the telephone (10.5%) and in-venue at fixed betting terminals (11.0%), at lower frequencies than the online modes of access.

### Predictors of In-Play Betting Behavior

An initial logistic regression was applied to assess which predictor variables statistically differentiated participants who bet in-play from those who did using the 12 predictor variables described in the Methods.

Income and country of birth predictor variables were removed from analysis due to lack of significance and poor contribution to model fit statistics. Ethnic background was also excluded from the final model because sparse data effects both reduced the model fit and led to uninterpretable odds ratios. As a robustness check, the model was run with these variables included, but the model fit improved with their removal.

The test of the final overall model with 9 predictors was significant, χ^2^ (22, *N* = 500) = 168.3, *p* < 0.001, indicating that, all together, these predictors reliably distinguished between in-play and non-in-play betting participants in the sample. The Hosmer and Lemeshow Test was not significant (*p* > 0.05), indicating a good model fit. Overall prediction success was 77.1%, with moderate predictive success for in-play betting participants (60.6%) and stronger accuracy for non-in-play betting participants (85.9%). The regression variables were assessed for multicollinearity using Variance Inflation Factor diagnostics, which were under 1.6 for all variables, well under the threshold of an indication of multicollinearity issues (37).

Table 3 displays regression coefficients, coefficient standard errors, Wald statistics, significance level, odds ratio, and 95% confidence intervals for each of the 10 predictor variables. Categorical variables used the following reference groups: gender (male), education level (post-graduate qualification), employment status (work full-time), language other than English spoken at home (yes), highest gambling frequency (at least once per day), and PGSI classification (score 8 or higher).

**Table 3:** Logistic regression results for characteristics differentiating participants who bet in-play vs. those who did not bet in-play (*N* = 500).

**Predictor** **variable**	**B**	**S.E. (B)**	**Wald**	**Significance** **level**	**Odds** **ratio**	**95% CI** **(lower)**	**95% CI** **(upper)**
Gender	0.170	0.254	0.447	0.504	1.185	0.721	1.948
**Age**	**−0.039**	**0.011**	**13.085**	**<0.001**	**0.962**	**0.942**	**0.983**
Education level			3.743	0.291			
University or college degree	0.514	0.380	1.826	0.177	1.672	0.793	3.525
Trade/technical diploma	0.019	0.428	0.002	0.965	1.019	0.440	2.358
Year 12 or equivalent	0.120	0.418	0.083	0.773	1.128	0.497	2.558
Employment status			7.215	0.065			
Work part-time or casual	−0.202	0.308	0.432	0.511	0.817	0.447	1.493
Non-salaried	−0.514	0.394	1.702	0.192	0.598	0.276	1.295
**Welfare recipient**	**−1.154**	**0.462**	**6.249**	**0.012**	**0.315**	**0.128**	**0.779**
Language other than English at home	−0.491	0.337	2.118	0.146	0.612	0.316	1.186
**Number of gambling behaviors**	**0.183**	**0.066**	**7.644**	**0.006**	**1.201**	**1.055**	**1.367**
**Age first gambled (ln)**	**1.387**	**0.450**	**9.512**	**0.002**	**4.003**	**1.658**	**9.666**
**Highest gambling frequency**			**8.109**	**0.017**			
**At least once per week**	**−0.777**	**0.295**	**6.950**	**0.008**	**0.460**	**0.258**	**0.819**
**At least once in the last 4 weeks**	**−0.991**	**0.417**	**5.637**	**0.018**	**0.371**	**0.164**	**0.841**
**PGSI classification**	**−1.036**	**0.258**	**16.177**	**<0.001**	**0.355**	**0.214**	**0.588**

*Significant predictors are identified in bold*.

Controlling for all other variables in the model, the significant predictors (α = 0.05) were: age, employment status (for Welfare recipient, compared to Work full-time), age first gambled, number of gambling behaviors, gambling frequency, and PGSI score.

## Discussion

This study makes a significant contribution by providing insight into the characteristics of those who place in-play bets, overcoming limitations of previous studies which focus on analyzing gambling behaviors without controlling for significant personal variables and betting across different modes and activities. The results of this study show that among the sample of participants who regularly gamble online, in-play betting is relatively common. Three in 10 participants had placed bets after an event had started, and this occurred mostly via online methods which are prohibited under Australian regulations. Demographic differences were found between those who placed bets in-play and those who did not: in-play bettors were more likely to be more highly educated, employed, younger, and from culturally and ethnically diverse backgrounds (albeit not country of birth). Individuals who received income from welfare sources including a pension, unemployment, or disability benefits, were less likely to bet in-play than respondents who work full time. As in-play betting was associated with younger age, however, this finding may reflect a likelihood of older participants to be retired. No specific differences were found in relation to gender although the different approached significance with a greater proportion of females engaged in in-play betting. The relationship between gender and in-play betting and gambling problems warrants additional investigation particularly as several previous studies have been based on almost entirely male samples (20, 25).

Those who placed bets in play were more involved in gambling overall in terms of frequency and number of activities. This is consistent with previous studies (15, 20, 25). Higher levels of problem gambling severity were observed among those who placed in-play bets, which is a novel finding as our results control for a greater range of relevant factors than previous research including individual characteristics, gambling behavior, and gambling history. Several of the characteristics of those who bet in-play are similar to the profile of Australians who use offshore (as opposed to only domestic) online gambling sites, suggesting there may be some confound or overlap given in-play betting is only available via offshore gambling sites (34). Our hypothesis was supported as after adjusting for gambling involvement, participants who had placed bets in-play were approximately three times more likely to be classified as having a gambling problem than those who had not placed this bet type, indicating an association between in-play betting and gambling problems. These findings are consistent with previous research (24) which is important as it demonstrates the consistency of findings across jurisdictions despite policy differences in prohibition and legalized in-play betting.

As with previous studies, our results are based on cross-sectional data and we cannot draw conclusions regarding causality. The structural characteristics of in-play betting mean that these bets require a rapid decision based on quick reactions to within-game events and are more similar to continuous and rapid gaming than most other forms of wagering which is typically discontinuous with low event frequency. These characteristics may make in-play betting more appealing and potentially problematic. For example, individuals with gambling problems are more likely to consume impulsively, using immediate forms of gambling in which the time period between bet and outcome is shorter (5, 27, 38). This is likely related to findings that higher trait impulsivity is common among those with gambling problems (39, 40). As such, online in-play betting products may be particularly harmful for individuals who are vulnerable to experiencing gambling problems.

In addition to the lack of evidence regarding causality, our methodology included other limitations. To be eligible to participate in the study, respondents had to have gambled online in the past month, meaning that respondents were likely more frequently engaged in gambling than the broader population of online gamblers. Further, the survey was described as a gambling study, making it more likely to catch the attention of potential respondents with a specific interest in gambling. As such, the results should be interpreted in relation to this specific sample of online gamblers rather than as an accurate level of gambling involvement or gambling problems among all those who have made in-play bets.

In terms of implications, our findings support the prohibition of online in-play betting in Australia based on the principle of limiting the availability of gambling products that are strongly associated with gambling-related harm. It is crucial to note that the association between in-play betting and gambling problems is independent of involvement in other gambling activities and is consistently found across jurisdictions regardless of policies to legalize or prohibit this gambling activity. The findings suggest that further regulatory attention needs to be paid to this gambling activity and efforts made to identify those who bet in-play to assess for gambling harms as well as to develop specific prevention interventions for in-play betting.

Since the time of data collection, efforts have been made in Australia to reduce the availability of and demand for offshore gambling sites, by which in-play betting can be accessed. The extent to which restricting in-play betting may encourage consumers to use offshore gambling sites should be continuously evaluated due to the risks associated with this activity. Further research on the mechanisms by which in-play betting may cause harm is warranted, including consideration of other gambling products that allow continuous bets to be placed within short decision periods, such as electronic gaming machines. How to differentiate between different variants of in-play betting and whether particular variants of in-play betting should be regulated, such as those involving longer time periods for decision-making, is a matter for further research.

## Data Availability Statement

The datasets presented in this article are not readily available because this would be a violation of the conditions of ethics approval. Requests to access the datasets should be directed to sally.gainsbury@sydney.edu.au.

## Ethics Statement

The studies involving human participants were reviewed and approved by University of Sydney Human Research Ethics Committee. The patients/participants provided their written informed consent to participate in this study.

## Author Contributions

SG and AB designed and conducted the survey. BA led the data analysis. SG led the manuscript preparation. BA and AB contributed to the manuscript editing and refining. All authors contributed to the article and approved the submitted version.

## Conflict of Interest

The authors declare that the research was conducted in the absence of any commercial or financial relationships that could be construed as a potential conflict of interest. The handling editor declared a past co-authorship with one of the authors AB.

## Footnotes

^1^Esports refers to professional video game tournaments.
